# Supplementary material for: Patterns of Intron Gain and Loss in Fungi
Source: PLoS Biol. 2004 Nov 30;2(12):e422. doi: 10.1371/journal.pbio.0020422 (PMC532390; doi:10.1371/journal.pbio.0020422)
Supplement: Table S1 — Also available at http://genes.mit.edu/NielsenEtAl/. (4.3 MB ZIP). [file pbio.0020422.st001.zip › NielsenEtAl/html/491.html]

AN3787.1.NCU00651.1.MG03598.1.FG00271.1


```
 CLUSTAL W (1.82) Multiple Sequence Alignments - Introns Inserted


Sequence 1: MG03598.1	458 aa
Sequence 2: FG00271.1	451 aa
Sequence 3: AN3787.1	441 aa
Sequence 4: NCU00651.1	382 aa
Alignment Length: 466 aa
Number Identitical Residues: 151 aa
Alignment Score (without introns) 8837


MG03598.1 	MASNINGPGPGDEEYGEEWAKDLRHRFERLLKTKRLNELD---RSRSRQGSPAPGTSSSS
NCU00651.1	----------------------------------------------------------MS
FG00271.1 	----MAGQHPPAGEYGEEWARDLRVQFEGLLRDKRMNDL----RNTSRQSSPSLGDQSPH
AN3787.1  	MADGKHHHSRRAPTTDAFDPAELTHAFEQLMRVKRFDRLLEKSRSPSRTQSPSP---SPY
          	 :..           .   . .     .     .  .   ..: . :   :.:    :  

MG03598.1 	PSASAGPSSTQPSSQIGSTPPS--YSSLRNLPKIPTPPAATDKESQKFRNLLITLSTTPT
NCU00651.1	NRDQKFDLHVIP--------------DLLSQPVVPEPPAQDDKEAQNFEKYLIAMSHIPL
FG00271.1 	MRSSPFPSDNRPSGSQGPALPS--YAALRHLPKIPSPPAAGDRDSQKFRNLLISLSLTPT
AN3787.1  	RSQPSHPSQALPATQPAQPQPSSQSPSLRNLPIVPYPPQ--DQNAFKFRNLLHVLSVTPT
          	           *: . . . .::. . *   * :* **   *::: :*.: *  :*  * 

MG03598.1 	KYENPGLLDEALQVIPLDRIYGEAEEETQVLQAQAESMGDGRKPEWGYQDCVIRALLR2W
NCU00651.1	NYENPGLLDEALQQIPLDRLSQEAEEEVELFQAKAASLG-RSKPEWSHQECMVRALLR2W
FG00271.1 	KYENPGLLDEALQTIPLDRIYGEAEEETQVLQAQAESMGDGRKPEWGYQDCVIRALLR2W
AN3787.1  	KYENPGLLDEALSLIPLDKLYSEADEECQIIQAQARSLK--RKPEWGYQDCVIRALLR2W
          	:***********. ****::  **:** :::**:* *:    ****.:*:*::***** *

MG03598.1 	FKRSFFTWINNPPCPVCMSPTEGKGKTAPTPEENACGALLVELYQCSSSHCGAYERFPRY
NCU00651.1	FRRSFFTFVNNPPCSECLSPTNKIRNVAPTPEERAHSATWVELYACVT--CGAYERFPRY
FG00271.1 	FKRSFFSWVNNPPCPSCLSPTIAQGMTAPTPEESACGALRVELYRCSAQHCGAYERFPRY
AN3787.1  	FKRSFFHWVNNPPCSRCLTPTIAHGRAPPTPDEAARGANRVELYRCADPSCGAYERFPRY
          	*:**** ::*****. *::**     ..***:* * .*  **** *    **********

MG03598.1 	SDVWRLLQTRRGRCGEWANCFSMLCRAVGGRVRWVWNAEDHVWTEIYSEHQKRWVHVDAC
NCU00651.1	TEAWQLLRVKRGRAGDFANVFTMLCRALDIRARWVWCQEDYLWTEIYSEHQQRWVHVDSC
FG00271.1 	GDVWRLLQTRRGRVGEWANCFSMLCRAVGGRVRWVWNAEDHVWTEVYSDHQKRWVHVDAC
AN3787.1  	SDVWQLLQTRRGRVGEWANCFSMFCRALGGRVRWVWNSEDYVWTEVYSEHQKRWIHVDAC
          	 :.*:**:.:*** *::** *:*:***:. *.****  **::***:**:**:**:***:*

MG03598.1 	EEAWDNPRLYTEG~WGKKMSYCVAFSIDGATDVTRRYVRK-SEFSLERNKCPEEVMLYIM
NCU00651.1	EEAWDMPHMYYKN~WGKKMSYVIAFSREGAVDVTRRYVGS-PDALLPRTRCPEGVLKFIM
FG00271.1 	EEAWDNPRLYAEG~WGKKMSYCIAFSIDGATDVTRRYVRK-NQHASERNRCPEEVLLYVM
AN3787.1  	EETWDQPRLYAEG1WGRKISYCIAFSIDGATDVTRRYVRSPAKHGAPRSRVPEEVLVWII
          	**:** *::* :. **:*:** :*** :**.******* .. .    *.: ** *: :::

MG03598.1 	QEIKNIRRANMNKD--ERFRLEKEDAREDRELRQYVVNSIAHAVTQLVPGVANGSGSSVS
NCU00651.1	EEITNLHRPKYAPDGETRLRLYREDVAEDVQLRSLWSATLEQSRR--LKAAAAAAARGGR
FG00271.1 	QEIKNMRRSNMNKD--ERFRLEKEDTREDKELRGYVVASIAQAVTDLVPGSPGGSNHTTA
AN3787.1  	QEIRKMRRENRPKE--EQRRLFKEDEREERELRMYTACALAAELKNLLP-----SNRSPS
          	:** :::* :   :   : ** :**  *: :**     ::     . :      :     

MG03598.1 	GSSEDAKLPAEQLGRQSGNAEWVAARGEGGRHHYPPGPGDGSHRREYR
NCU00651.1	SSPDNKSGANMMGSPATGDIKRPIPEDAPVPDVPSLWPTYGP------
FG00271.1 	SGS-DTKLPAEQPGRQTGTSEWLTAQQQQSHSRYQQ-PRDPSHRRPLP
AN3787.1  	ARPDEVKVPQT----AEPQAAWNNSRQRSGHS-----GPDGSQGDR--
          	. ..: . .               ..               .
```
